# Adding to the burden: the tendency to resonate with others’ stress is linked to higher PTSD symptom severity in individuals with war-related trauma

**DOI:** 10.1038/s41398-025-03548-4

**Published:** 2025-08-30

**Authors:** Christiane Wesarg-Menzel, Mathilde Gallistl, Michael Niconchuk, Veronika Engert

**Affiliations:** 1 Social Stress and Family Health Research Group, Max Planck Institute for Human Cognitive and Brain Sciences, Leipzig, Germany; 2 Institute for Psychosocial Medicine, Psychotherapy and Psychooncology, Jena University Hospital, Friedrich-Schiller University, Jena, Germany; 3 Beyond Conflict, Boston, MA USA; 4 German Center for Mental Health (DZPG), partner site Halle-Jena-Magdeburg, Jena, Germany; 5 Center for Intervention and Research on adaptive and maladaptive brain Circuits underlying mental health (C-I-R-C), Halle-Jena-Magdeburg, Jena, Germany

**Keywords:** Diagnostic markers, Physiology

## Abstract

Many refugees experience multiple traumatic events, which set them at increased risk to develop post-traumatic stress disorder (PTSD). To refine interventions aimed at improving refugees’ mental health, a better understanding of the factors modulating vulnerability to war-related trauma is needed. In the present study, we focused on stress resonance as a potential vulnerability factor. Stress resonance reflects the empathic sharing of others’ subjective and physiological stress experience. Sixty-seven participants who came from Arabic-speaking countries and had entered Germany as refugees or migrants took part in an empathic stress test, in which they observed a native German speaker undergo a psychosocial laboratory stressor. Meanwhile, different stress markers (subjective stress, heart rate, heart rate variability, and cortisol release) were simultaneously captured in the stressed targets and passive observers. Moderation analyses did not support our hypothesis that the extent to which someone resonates with others’ stress is a vulnerability factor in the development of PTSD symptoms after trauma exposure. Rather, higher levels of subjective and autonomic stress resonance were directly related to PTSD symptom severity when controlling for sex, age, and trauma exposure. Our findings suggest that heightened stress resonance may constitute a malleable correlate of PTSD symptoms rather than a trait modulating health risk. In the future, efforts should be made to test whether individuals with a history of war-related trauma would benefit from interventions aimed to reduce the tendency to excessively share others’ stress.

During interactions, the perception of another individual’s state automatically stimulates a corresponding representation in the observer, leading to common physiological states in which the observer unintentionally matches the emotion, facial expression, or other behaviors of the interaction partner [1]. This physiological linkage between individuals has been studied under the terms “empathy”, “bio-behavioral synchrony”, or “resonance”, and is thought to facilitate social connection and coordination [1–3]. Although the adaptive value of resonating with others has been shown in non-threatening contexts such as parent-child interactions during play [4–6], resonance may come at the expense of health in the context of stress or other adverse experiences [3, 7]. This is of particular relevance for individuals who are exposed to others’ stress and suffering on a daily basis, as is the case for individuals living in or fleeing from areas of war.

Due to war and persecution, the number of refugees has shown a steady increase in the past decades, reaching an estimate of 36.4 million refugees at mid-2023 [8]. Traumatic events that refugees are exposed to are highly prevalent and diverse, ranging from lack of accommodation or necessities, to experiencing or witnessing violence, torture, and loss of loved ones [9, 10]. With the settlement in a new country, the accumulation of stressful experiences often continues due to difficulties in proving asylum claims and integrating in a new society [11, 12]. These experiences set refugees at higher risk to develop post-traumatic stress disorder (PTSD), which ranges with a prevalence of around 31.5% among the most commonly diagnosed psychiatric disorders in refugees [13].

War-related traumatic experiences are inherently embedded in a social context. Next to their own suffering, refugees are confronted with the suffering of family members and friends, as well as of strangers in their surroundings. Hence, apart from being victims of trauma inducing firsthand stress, refugees likely witness the trauma of others [14], which can induce additional second-hand (empathic) stress [3]. Importantly, the mere observation of another individual experiencing stress can trigger a full-blown stress response in the observing individual, apparent through activation of the autonomic nervous system (ANS) and hypothalamic-pituitary-adrenal (HPA) axis [15, 16]. While prolonged exposure to firsthand stress can lead to dysregulation of these neurobiological stress systems [17], such effects may be exacerbated in individuals with a strong tendency to resonate with others’ stress. In turn, dysregulation of the stress systems has been shown to underlie heightened vulnerability to psychopathology after trauma [18, 19]. Transferred to the reality of the lives of refugees, this would imply that despite being exposed to similar first- and second-hand traumatic experiences, one individual may be more likely to develop mental health problems than another based on inter-individual differences in the tendency to resonate with others’ stress.

Previous research has distinguished stress resonance from vicarious stress as two possible response patterns to observing another individual under stress: Vicarious stress refers to an observer’s own stress response *irrespective* of whether the target directly exposed to acute stress shows a stress response. In contrast, stress resonance is present when the observer’s stress response is *proportional* to the one of the acutely stressed target [16]. Whereas vicarious stress reactivity is thought to reflect an individual’s general stress sensitivity, stress resonance is closely linked conceptually and empirically to empathy [3, 16, 20]. While previous studies have shown that stress sensitivity is an important vulnerability factor underlying heightened risk for the development of psychopathology [21, 22], we here turn attention to stress resonance as a potential vulnerability factor in the context of trauma. Indirect evidence for this hypothesis stems from a study in disaster volunteers, in which those with a stronger tendency to feel discomfort and anxiety in response to others’ distress (reflecting trait levels of empathy) experienced higher increases in depression, anger, and fatigue from pre- to post-relief work [23]. Examining the role of stress resonance in the etiology of PTSD symptoms after trauma exposure bears clinical implications, as it could inform efforts to advance intervention research dedicated to improving refugees’ mental health.

Based on a sample of refugees and migrants from Syria and other Arabic-speaking countries, we examined in the current work whether stress resonance would constitute a vulnerability factor for developing PTSD symptoms after war-related trauma. Next to subjective stress resonance, we focused on three different markers of the stress systems: (1) cortisol as a neuroendocrine marker of the HPA axis, as well as (2) heart rate and (3) heart rate variability as physiological markers of sympathetic and parasympathetic nervous system (SNS, PNS) functioning. As a strong paradigm that reliably triggers stress resonance even in strangers, we used the empathic Trier Social Stress Test (TSST [16, 24]). In this paradigm, native Arabic-speaking participants watched native German-speakers undergo a standardized psychosocial stress test. The different stress markers were simultaneously captured in the stressed targets and passive observers. In a previous data set stemming from the same study, we found a moderate positive association between trauma exposure and PTSD symptoms [25]. In the present work, we draw on data from a subsample of this earlier study. Based on the above literature, we expected that stress resonance would moderate the link between trauma exposure and PTSD symptomatology, such that the positive association between trauma and PTSD symptoms would be stronger in individuals exhibiting higher stress resonance.

## Methods

### Participants

Our sample consisted of *N* = 66 targets (55 females) taking part in a stress test, and *N* = 67 observers of the opposite sex (11 females) watching the targets during the stress test (opposite-sex dyads were chosen as per our usual approach [16]). In the first testing session, two observers simultaneously watched one target. Given implementation of COVID-19 contact restrictions after testing of this first triad, all subsequent testing sessions were executed in a one-to-one observation set-up (i.e., dyads). Targets were German (age *M* = 26.0 years, *SD* = 5.22 years), and observers Arabic native speakers (age *M* = 28.7 years, *SD* = 4.85 years). Of the observers, 35 had entered Germany as refugees (age *M* = 30.4 years, *SD* = 4.59 years) and 32 as migrants (age *M* = 26.9 years, *SD* = 4.53 years). The majority of refugees and migrants came from Syria (88.6 and 71.9%, respectively; see Table S1 for countries of origin).

Participants were recruited through social media advertisement and by distributing flyers. Trained students conducted a structured telephone screening to determine participants’ eligibility. All participants needed to be aged between 20 and 40 years. Targets were required to speak German as the native language. Observers needed to speak Arabic as the native language and German at an intermediate (B1) level, come from an Arabic-speaking country of origin, and live for at least six months in Germany. Within observers, we further differentiated between refugees and migrants based on definitions of UNHCR [8]: Individuals who had been *forced* to flee their home countries because of conflict or persecution were considered as refugees. In contrast, individuals who had *chosen* to leave their home to work, study, or join family in a new country were considered as migrants. Refugees were included if they reported a war-related trauma (i.e., they had fled war, violence, or persecution) in the absence of other major trauma (e.g., child maltreatment, severe accident; queried based on a list of traumatic events compiled by the authors). In contrast, migrants were supposed to be free of any major traumatic experience.^1^ Given known effects of weight on autonomic activity [28], an additional exclusion criterion for participants of both target and observer groups was a BMI of less than 18 or more than 30. We further excluded participants who reported chronic illnesses, psychiatric disorders (e.g., anxiety disorders), or the regular use of medication, hormone-based birth control, or other substances (e.g., alcohol, drugs) known to affect HPA axis or autonomic activity [29, 30]. Refugees were not excluded if they had a diagnosis of PTSD or depression during the last two years. To facilitate recruitment, we further decided to include occasional and regular cigarette smokers in the observer group due to high prevalence of smoking in many Middle Eastern countries [31].

### Procedure

The study was approved by the Ethics Board of the medical faculty of Leipzig University, Germany (ethics number: 405/18-ek). Observers came to the lab for a first visit lasting about 2.5 h, in which they provided informed consent, filled out online questionnaires, and completed an empathy task (EmpaToM [32]; not subject to the current manuscript). For a second lab visit lasting about 4.5 h, observers were invited back together with an unfamiliar target person of opposite sex. This visit was scheduled in the early afternoon to control for variation in diurnal cortisol secretion [33].

Upon arrival at the laboratory, targets and observers were placed in separate rooms. Participants first underwent a rapid drug test to screen for recreational drug use. To equalize blood sugar levels, they were offered a snack and a glass of juice. To avoid saliva sample contamination, participants were instructed to not eat or drink anything other than water throughout the remainder of the testing session. Then, participants were equipped with a chest belt recording an electrocardiogram (ECG) to measure SNS and PNS activity. After a resting period of 40 min, participants were guided into either the testing or the adjacent observation room. While targets attended the TSST [24], observers passively watched the procedure (preparatory anticipation and stress phase) through a one-way mirror. Targets were aware of being observed during their performance, although they did not know by whom. After the TSST, target and observer separately left their rooms, and rested alone during the 60 min recovery phase.

The TSST is a standardized laboratory paradigm that reliably elicits social-evaluative stress [34, 35]. It consists of a preparatory anticipation phase (5 min), followed by a video-recorded mock job interview (5 min) and a mental arithmetic task (5 min) in front of an evaluation committee. Given safety rules due to the COVID-19 pandemic, for 60.6% of the testing sessions, the committee members were sitting in a separate room from the participant. Their image was projected via live video feed at large-scale on the wall facing the target. During the TSST instruction period, the committee members shortly entered the TSST testing room to demonstrate their actual presence in the laboratory and thus the realness of the video situation.

**Fig. 1:**
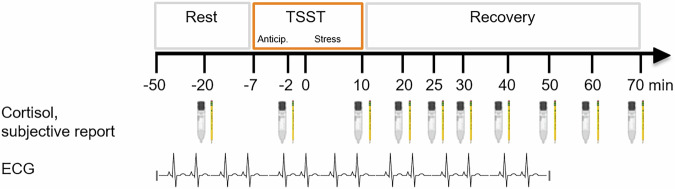
Assessment timeline. Anticip. anticipation phase.

Throughout the testing period, ten ratings of subjective stress experience and ten cortisol samples were collected simultaneously from targets and observers. In addition, a continuous ECG was collected from 50 min prior to 50 min post stressor onset (for assessment timeline, see Fig. 1). Participants received financial compensation for study participation. The study was performed in agreement with the Declaration of Helsinki. All participants gave their written informed consent and could withdraw from the study at any time.

### Measures of acute stress reactivity

#### Subjective stress experience

In both targets and observers, we assessed participants’ subjective stress experience at ten times throughout the test session using a 7-point Likert scale asking for the level of currently experienced stress (“How stressed do you feel at this moment?”). Assessments took place 20 min prior to stressor onset (baseline), after stress anticipation (2 min prior to stressor onset), and at 10, 20, 25, 30, 40, 50, 60 and 70 min after stressor onset (see Fig. 1).

#### Salivary cortisol

In parallel to the assessments of subjective stress experience, participants collected ten salivary samples for cortisol analysis using Salivettes (Sarstedt, Nümbrecht, Germany; Fig. 1). Participants placed the collection swab in their mouth for 2 min and refrained from chewing. Salivettes were stored at −20 °C until analysis. Cortisol levels were assessed using a time-resolved fluorescence immunoassay with intra- and inter-assay variabilities of less than 10 and 12%, respectively [36].

#### Heart rate and heart rate variability

All participants wore a Zephyr Bioharness 3 chest belt (Zephyr Technology, Annapolis, Maryland, USA), which recorded a continuous ECG at a frequency of 250 Hz for altogether 100 min (from −50–+50 min relative to stressor onset). A trained student manually corrected artifacts in the ECG raw data using python-based in-house software. Two students re-checked all corrections made. For further analysis, each ECG recording was split into 5-min timeframes related to specific phases of the testing protocol (Fig. 1). The baseline phase was represented by timeframes 1 and 2 (from −40 to −30 min), the anticipation phase by timeframe 3 (from −7–−2 min), the stress phase by timeframes 4 and 5 (from 0–+10 min), and the recovery phase by timeframes 6–12 (from +15–+50 min). For each timeframe, we calculated averages for heart rate (HR) and heart rate variability (HRV; specifically the root mean of the square successive differences [RMSSD]) per participant using the python package “hrv-analysis” [37]. To ensure sufficient data quality, we excluded timeframes in which more than 10% of the recording had to be cut.

### Questionnaires

#### Trauma

Trauma was measured with the official Arabic (“Iraqi”) version of the trauma events section of the Harvard Trauma Questionnaire (HTQ [26, 38]). Participants indicated whether they had experienced each of 42 traumatic events (e.g., oppression, imprisonment, combat exposure) before coming to Germany. Trauma scores were calculated by a sum of “yes” responses. In our study, the trauma events section of the HTQ demonstrated excellent reliability (Cronbach’s α = 0.92).

#### PTSD symptoms

We assessed PTSD symptom severity with the first 16 items of the trauma symptom section of the HTQ [38], which correspond to symptoms of PTSD according to the DSM-IV. Participants indicated the degree to which they were distressed in the past week by trauma symptoms such as “feeling detached or withdrawn from people” or “trouble sleeping” on a Likert scale ranging from 1 (“not at all”) to 4 (“extremely”). Higher mean scores indicated more severe PTSD symptoms. The HTQ manual recommends a cut-off score of 2.5 to identify clinically significant levels of PTSD [26], which was the case in five observers. Reliability was good (Cronbach’s α = 0.89).

#### Personal distress

As a specific facet of empathy potentially linked to stress resonance, we assessed trait personal distress based on the Interpersonal Reactivity Index (IRI [39]). The IRI was translated into Arabic by a native, bilingual speaker and back-translated by another bilingual individual to ensure linguistic equivalence [40]. The personal distress scale measures self-oriented feelings such as discomfort, anxiety and worry when being confronted with others’ negative emotional states or situations. It consists of seven items rated on a Likert scale from 1 (“does not describe me well”) to 5 (“describes me very well”), aggregated to a sum score. In the current study, reliability was relatively poor (Cronbach’s α = 0.49).

### Data analysis

#### Data processing and computation of stress measures

To approach normal distribution, cortisol, HR, and HRV data were log-transformed and subsequently winsorized to three standard deviations [29]. For all participants and stress markers, we calculated change scores from baseline to stress peak. While change scores indicated stress reactivity in targets, they reflected vicarious stress (i.e., stress reactivity independent of target stress) in observers.

For cortisol, the baseline level (sample 1 at −20 min) was subtracted from the individual peak level chosen from samples at +10, +20, +25, +30, or +40 min (samples 3−7), since cortisol typically peaks within this timeframe after stressor onset [41, 42]. A significant physiological stress response was defined as a cortisol increase of at least 1.5 nmol/l above baseline levels [43]. For HR, the mean of timeframes 1 and 2 (baseline) was subtracted from the maximum of timeframes 3 (anticipation), 4 and 5 (stress task). For HRV, the minimum of timeframes 3 − 5 was subtracted from the mean of timeframes 1 and 2 (baseline). This was done because other than HR, HRV *decreases* with stress experience [44]. Hence, higher change scores indicated stronger increases in cortisol and HR, and stronger decreases in HRV in response to stress. If HR / HRV data was only available for the anticipation phase, but missing for the stress phase, we refrained from computing a change score, as it was impossible to verify whether the anticipation sample represented peak reactivity. For subjective stress, we subtracted the baseline rating (at −20 min) from the peak level chosen from ratings at −2 and +10 min. Since baseline levels can affect the reactivity of physiological parameters (law of initial value [45]), we adjusted cortisol, HR, and HRV change scores for baseline levels by extracting the standardized change score residuals from a regression model. Residualized change scores were chosen rather than percentage change scores as the latter are highly sensitive to baseline levels (i.e., particularly small baseline levels can yield exaggerated percentage changes).

To gain a measure of physiological and subjective stress resonance, we subtracted observer change scores from the corresponding target’s change scores, and computed an absolute value of this difference. By doing so, we obtained a measure of how closely the observer’s stress reactivity resembled the one of the target. The absolute difference score was multiplied by (−1), such that higher values indicated higher stress resonance.

#### Missing data

Data on vicarious stress and stress resonance was complete for subjective stress and cortisol. Due to technical issues, HR and HRV data was missing for two observers and two targets (of whom one observer and one target belonged to the same dyad). In addition, HR and HRV data quality was too low (i.e., >10% needed to be cut) in one observer during both baseline and stress timeframes, and in one target during both stress timeframes, precluding the computation of change scores. Thus, overall, data on HR and HRV vicarious stress was missing for three observers, and data on HR and HRV stress resonance was missing for five observers.

#### Descriptives

We first examined the number of targets and observers showing a significant cortisol stress response (cortisol increase >1.5 nmol/L from baseline [43]). To detect possible differences in our variables of interest between refugees and migrants, we compared both groups with regard to their vicarious stress responses and stress resonance across all four stress markers, as well as regarding trauma experience and PTSD symptoms.

#### Logistic regression modeling

Regression analyses were carried out to test for the relation between stress resonance and PTSD symptoms. We initially planned to conduct multiple linear regression analyses, but because assumptions were violated in most models (e.g., presence of heteroscedasticity/unequal variances of residuals), we applied multiple logistic regressions. To this end, we created a dichotomous outcome measure of PTSD symptoms above and below the sample mean. All continuous predictors were *z*-standardized to handle possible issues of multicollinearity. For each stress marker, the base model included trauma and the covariates sex and age as predictors of PTSD symptoms. In a second step, we tested whether adding stress resonance (operationalized as the absolute value of the difference between target and observer change scores) and its interaction with trauma would improve model fit, which was evaluated by comparing both models using analysis of variance. This procedure resulted in one model per stress marker (subjective stress, cortisol, HR, HRV), yielding four final models. Given multiple testing, we applied Bonferroni correction, such that *p*-values were compared to a significance threshold of α = 0.0125.

#### Sensitivity analyses

Due to significant correlations between observers’ stress resonance and vicarious stress for subjective stress, HR, and HRV, we re-computed analyses by introducing vicarious stress instead of stress resonance as a moderator to the regression models. Further, since the drug screening conducted before the TSST was positive for *n* = 4 targets (6.1%) and *n* = 3 observers (4.5%), we re-computed analyses by excluding the seven dyads in which either target or observer had a positive test result.

## Results

### Descriptives

**Fig. 2:**
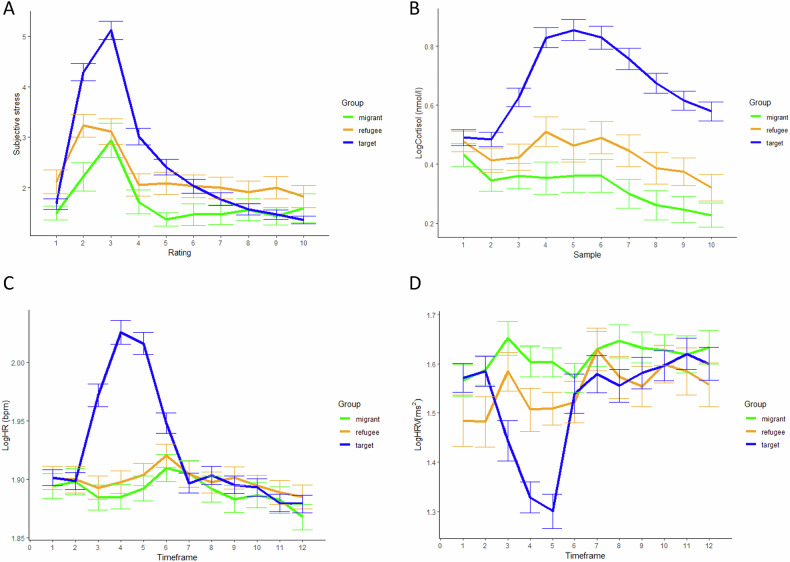
Mean levels of subjective stress, cortisol, HR, and HRV throughout the testing session per group. **A** Subjective stress. **B** Cortisol. **C** Heart rate. **D** Heart rate variability. LogCortisol logarithmized cortisol, LogHR logarithmized heart rate, LogHRV logarithmized heart rate variability.

Concerning firsthand stress, 47 out of 66 targets (71.2%) demonstrated a significant physiological stress response, defined as a cortisol increase of > 1.5 nmol/l over baseline levels [43]. Among the 67 observers, 12 (17.9%; 5 refugees, 7 migrants) showed a physiologically significant increase in cortisol levels after passively watching a target undergoing the TSST. The difference in responder rates between migrants and refugees was not significant, χ²(1,67) = 0.65, *p* = 0.42. Intriguingly, 9 out of 67 observers (13.4%) showed a higher cortisol change score than the according target, indicating that the observer’s vicarious stress response in some cases surpassed the target’s firsthand response. Within observers, non-smokers (*N* = 37) did not significantly differ from smokers (*N* = 30) with regard to vicarious stress across the four stress markers (i.e., subjective stress, cortisol, HR, and HRV; see Table S2). Further, taking the four stress markers into consideration, there were no significant differences between refugees and migrants in either vicarious stress or stress resonance (see Table 1). Average subjective stress, cortisol, HR, and HRV levels throughout the testing session per group are shown in Fig. 2. Analyses of variance showed significant group differences for baseline subjective, but not physiological stress levels (see Table S3). A post-hoc Tukey test revealed that refugees reported higher levels of subjective stress at baseline than migrants (*p* = 0.037), while targets did not differ in their baseline subjective stress from migrants (*p* = 0.680) or refugees (*p* = 0.104; see Table S3). Refugees reported more traumatic events and higher levels of PTSD symptoms than migrants (see Table 1). A full correlation matrix is shown in Table 2.

**Table 1:** Comparison of refugees and migrants on trauma, PTSD symptoms, and empathic stress measures.

	Refugees *N* = 35	Migrants *N* = 32	
	*M* (*SD*)	*M* (*SD*)	*t, p*
Trauma	16.23 (7.61)	8.41 (7.37)	*t* = −4.27, *p* < 0.001***
PTSD symptoms	1.89 (0.49)	1.52 (0.46)	*t* = −3.13, *p* = 0.003**
**Vicarious stress**			
Δ Subjective stress	1.60 (1.4)	1.62 (1.79)	*t* = 0.06, *p* = 0.949
Δ Cortisol^a^	0.09 (0.24)	0.03 (0.24)	*t* = −1.09, *p* = 0.282
Δ HR^a^	0.01 (0.03)	0.00 (0.03)	*t* = −0.94, *p* = 0.353
Δ HRV^a^	0.01 (0.17)	0.02 (0.12)	*t* = 0.19, *p* = 0.850
**Stress resonance**			
Subjective stress resonance	2.43 (1.52)	2.66 (1.66)	
Cortisol resonance	0.40 (0.28)	0.49 (0.29)	*t* = 1.28, *p* = 0.230
HR resonance	0.14 (0.06)	0.12 (0.07)	*t* = −0.72, *p* = 0.476
HRV resonance	0.30 (0.24)	0.32 (0.23)	*t* = 0.33, *p* = 0.742

^a^Change scores based on log-transformed and winsorized data.

**p < 0.01, *** *p* < 0.001.

**Table 2:** Full correlation matrix.

	SexO^a^	AgeO	Trauma	PTSD	PD	SSO	SSRes	SST	CO	CRes	CT	HRO	HRRes	HRT	HRVO	HRVRes
AgeO	−0.04															
Trauma	−0.28*	0.14														
PTSD	0.23	0.26*	0.40***													
PD	0.25*	−0.10	−0.02	0.39**												
SSO	0.06	0.02	0.02	0.24*	0.40***											
SSRes	0.28*	−0.07	0.02	0.30**	0.32**	0.49***										
SST	−0.41***	−0.06	0.06	−0.19	−0.09	0.05	−0.54***									
CO	−0.05	0.10	−0.07	0.14	0.07	−0.10	0.02	−0.18								
CRes	−0.13	0.19	0.04	0.05	−0.06	0.11	0.02	−0.09	0.23							
CT	0.22	−0.14	−0.13	−0.14	0.12	−0.24	−0.06	−0.05	−0.10	−0.73***						
HRO	−0.33**	0.02	0.14	−0.02	0.10	0.09	−0.01	0.13	0.15	0.16	−0.14					
HRRes	−0.12	−0.25	0.02	0.13	0.13	−0.02	−0.07	0.18	0.11	0.25	−0.38**	0.54***				
HRT	−0.05	0.29*	0.06	−0.18	−0.09	0.08	0.10	−0.16	−0.05	−0.21	0.37**	−0.15	−0.91***			
HRVO	−0.18	−0.09	0.09	0.02	0.22	0.10	0.03	−0.05	0.15	−0.11	0.05	0.50***	0.18	0.03		
HRVRes	−0.10	−0.25	0.13	0.25*	0.33**	0.17	0.08	0.02	0.14	0.21	−0.34**	0.43***	0.70***	−0.61***	0.55***	
HRVT	−0.03	0.25*	−0.06	−0.28*	−0.26*	−0.09	−0.02	−0.09	−0.05	−0.30*	0.40**	−0.22	−0.74***	0.76***	−0.09	−0.87***

*AgeO* age of observer, *CO* cortisol reactivity of observer, *CRes* cortisol resonance, *CT* cortisol reactivity of target, *HRO* heart rate reactivity of observer, *HRRes* heart rate resonance, *HRT* heart rate reactivity of target, *HRVO* heart rate variability reactivity of observer, *HRVRes* heart rate variability resonance, *HRVT* heart rate variability reactivity of target, *PD* personal distress, *PTSD* post-traumatic stress disorder symptoms, *SexO* sex of observer, *SSO* subjective stress of observer, *SST* subjective stress of target, *SSRes* subjective stress resonance.

^a^Sex coded as 0 = male, 1 = female.

*p < 0.05, ** *p* < 0.01, *** *p* < 0.001.

### Logistic regression analyses

Table 3 presents the base models with trauma, sex, and age as predictors of PTSD symptom severity (divided into above vs. below average), as well as four extended models in which stress resonance (subjective, cortisol, HR, and HRV) was added. In the base model, trauma exposure and female sex were the most important risk factors of PTSD symptoms. In the extended models, increasing resonance in the markers of subjective stress, HR, and HRV, but not cortisol, were associated with increasing odds for reporting above average numbers of PTSD symptoms (significance evaluated against Bonferroni-corrected threshold of α = 0.0125).

**Table 3:** Logistic regression analyses predicting PTSD symptoms.

	*B (SE)*	*p*	*OR (95% CI)*			
**Base model (*N*** = **67)**						
Intercept	−0.65 (0.32)	0.042				
Trauma	0.81 (0.31)	0.009*	2.25 (1.26, 4.34)			
Sex	3.03 (0.99)	0.002*	20.61 (3.58, 193.02)			
Age	0.62 (0.32)	0.054	1.86 (1.02, 3.66)			
*R*^2^ (Nagelkerke) = 0.336. Model *X*^2^(3) = 19.44, *p* < 0.001*						
**Models including stress resonance (*N*** = **67)**						
	**Subjective stress**			**Cortisol**		
	*B (SE)*	*p*	*OR (95% CI)*	*B (SE)*	*p*	*OR (95% CI)*
Intercept	−0.71 (0.36)	0.051		−0.66 (0.32)	0.042	
Trauma	0.76 (0.35)	0.029	2.13 (1.11, 4.43)	0.85 (0.32)	0.008*	2.34 (1.29, 4.63)
Sex	2.60 (1.05)	0.013	13.42 (2.06, 144.13)	3.13 (1.01)	0.002*	22.83 (3.83, 225.83)
Age	0.77 (0.37)	0.035	2.16 (1.11, 4.77)	0.53 (0.33)	0.112	1.70 (0.90, 3.39)
Stress resonance	1.07 (0.39)	0.006*	2.93 (1.45, 6.77)	0.36 (0.31)	0.243	1.43 (0.80, 2.72)
Trauma*Stress resonance	0.32 (0.41)	0.433	1.37 (0.63, 3.18)	0.12 (0.27)	0.657	1.13 (0.66, 2.01)
Model fit	*R*^2^ (Nagelkerke) = 0.485. Model *X*^2^(5) = 30.21, *p* < 0.001*			*R*^2^ (Nagelkerke) = 0.363. Model *X*^2^(5) = 21.25, *p* < 0.001*		
Delta model fit	*X*^2^(2) = 10.77, *p* = 0.005*			*X*^2^(2) = 1.82, *p* = 0.404		
**Base model based on sub-sample with complete autonomic resonance data (*N*** = **62)**						
	*B (SE)*	*p*	*OR (95% CI)*			
Intercept	−0.56 (0.32)	0.082				
Trauma	0.75 (0.31)	0.016	2.11 (1.18, 4.04)			
Sex	2.59 (1.02)	0.011*	13.38 (2.18, 130.37)			
Age	0.58 (0.31)	0.066	1.78 (0.99, 3.45)			
*R*^2^ (Nagelkerke) = 0.288. Model *X*^2^(3) = 15.04, *p* = 0.002*						
**Models including stress resonance (*N*** = **62)**						
	**HR**			**HRV**		
	*B (SE)*	*p*	*OR (95% CI)*	*B (SE)*	*p*	*OR (95% CI)*
Intercept	−0.61 (0.35)	0.084		−0.73 (0.37)	0.047	
Trauma	0.73 (0.34)	0.031	2.09 (1.10, 4.30)	0.65 (0.34)	0.058	1.91 (1.01, 3.92)
Sex	3.09 (1.14)	0.007*	22.04 (2.97, 280.32)	3.52 (1.26)	0.005*	33.65 (3.70, 571.64)
Age	0.90 (0.37)	0.016	2.45 (1.25, 5.50)	1.11 (0.43)	0.010*	3.04 (1.41, 7.76)
Stress resonance	0.93 (0.36)	0.010*	2.53 (1.30, 5.51)	1.05 (0.41)	0.010*	2.87 (1.39, 7.07)
Trauma*Stress resonance	0.51 (0.34)	0.131	1.66 (0.89, 3.45)	0.26 (0.35)	0.452	1.30 (0.65, 2.60)
Model fit	*R*^2^ (Nagelkerke) = 0.432. Model *X*^2^(5) = 24.23, *p* < 0.001*			*R*^2^ (Nagelkerke) = 0.437. Model *X*^2^(5) = 24.60, *p* < 0.001*		
Delta model fit	*X*^2^(2) = 9.20, *p* = 0.010*			*X*^2^(2) = 9.57, *p* = 0.008*		

*HR* heart rate, *HRV* heart-rate variability (indexed via RMSSD).

*p < 0.0125.

### Sensitivity analyses

We re-ran analyses by introducing vicarious stress instead of stress resonance as a moderator to the regression models. In all four models on subjective stress, cortisol, HR, and HRV, vicarious stress was neither a significant main predictor of PTSD symptoms nor a significant moderator of the link between trauma and PTSD symptoms when applying a Bonferroni-corrected threshold of α = 0.0125 to evaluate significance (see Table S4). In a second sensitivity analysis, we excluded seven dyads in which either target or observer had a positive drug screening on the day of stress testing (see Table S5). Based on the reduced data set, the interpretation of results remained unchanged for subjective stress resonance. While HR stress resonance as well as HRV stress resonance were significant predictors of PTSD symptoms at an uncorrected threshold of α = 0.05, they did not remain significant when applying a Bonferroni-corrected threshold of α = 0.0125.

## Discussion

As a proxy for the role of empathy in the risk for PTSD, we assessed stress resonance in a sample of individuals from Arabic-speaking countries with varying levels of war-related trauma exposure. We did not find evidence to support our hypothesis that the extent to which someone resonates with others’ stress is a vulnerability factor in the development of PTSD symptoms after trauma exposure. Rather, higher levels of subjective and autonomic stress resonance were directly related to PTSD symptom severity when controlling for sex, age, and trauma exposure. Our findings hence suggest that heightened empathic stress resonance may constitute a correlate of PTSD symptoms rather than a stable trait modulating health risk. We further observed that subjective and HRV stress resonance were moderately related to trait levels of empathy, as assessed with the personal distress scale of the IRI [39]. In other words, individuals who indicated to have the tendency to feel uneasiness or worry when exposed to the negative experiences of others also showed higher levels of stress resonance in a naturalistic situation in which they watched a stranger exposed to acute stress.

Given the scarcity of studies on stress resonance in prior trauma and PTSD research, we discuss our findings primarily against the backdrop of studies assessing personal distress, which correlated with stress resonance in our study. In line with our finding of higher subjective and autonomic stress resonance being linked to higher PTSD symptom severity, disaster workers who reported higher trait levels of personal distress also reported higher post-traumatic stress responses and general psychological distress after engaging in relief activities following a typhoon [46]. Moreover, Parlar et al. [47] showed that women with PTSD related to childhood trauma reported higher levels of personal distress compared to healthy control women. Higher levels of personal distress were further observed in individuals with PTSD related to various other types of trauma (man-made trauma, accidental trauma or natural catastrophes) compared to a non-traumatized control group [48]. In this specific study, the PTSD group further showed lower levels of empathic resonance measured as contagion to yawning and laughing than the control group [48]. Although this finding may seem contradictory to our observed positive association between PTSD symptom severity and resonance, the inconsistency may be explained by the context represented in the different tasks. While laughing and yawning are positive and relatively neutral stimuli, respectively, we focused on the negative context of stress. Taken together, it may be that PTSD symptoms relate to higher resonance in negative settings, and lower resonance in positive settings. In support of this assumption, previous research has shown that emotional deficits associated with PTSD include a hyper-responsivity to negative stimuli and an increased threshold for responsivity to positive stimuli [49]. Relatedly, young adults reporting higher trait personal distress experienced higher levels of negative and lower levels of positive emotions in the initial stage of the COVID-19 pandemic [50].

Apart from PTSD, higher levels of personal distress have been observed in the context of various other psychiatric disorders, including major depressive disorder, bipolar disorder, borderline personality disorder, and schizophrenia [51–55]. Given the link between personal distress and stress resonance observed in our study, we speculate that the empathic tendency to pick up on and resonate with the adverse states of others may portray an unspecific symptom across psychiatric disorders.

In our study, stress resonance and PTSD symptoms were captured following trauma exposure. We have previously shown that exposure to trauma predicted PTSD symptoms in a larger data set stemming from the same study [25]. Although it would be plausible to assume that trauma exposure could have also modulated stress resonance, participants’ stress resonance was unrelated to their trauma exposure. It hence seems that alterations in stress resonance were not a direct reaction to the trauma, but may have been provoked by common sequelae of trauma implicated in PTSD development. These can include alterations in attentional processes such as hypervigilance, reduced attentional control, difficulty to disengage attention from threat, and inhibition problems [56–59]. In addition, individuals with PTSD often show difficulties in emotion regulation [60, 61], which may predispose them to experience heightened personal distress when being confronted with others’ negative experiences [62]. Further support for a close link of personal distress with psychopathology rather than trauma exposure per se stems from a study by Dittrich et al. [53] who observed no significant association between early-life maltreatment and personal distress when controlling for participants’ diagnosis of major depressive or borderline personality disorder. In contrast, a recent study observed that increased personal distress in adults was associated with higher levels of childhood maltreatment [63]. Yet, as acknowledged by the authors, participants were not excluded based on any psychopathologies they may have had, which prevented testing for a potential confounding of symptoms of psychopathology in explaining the link between trauma and personal distress.

Since we assessed stress resonance and PTSD symptoms simultaneously, we cannot establish their temporal sequence or causal relationship. It is possible that alterations in stress resonance preceded the development of PTSD symptoms. Alternatively, both could have emerged concurrently, or alterations in stress resonance might have been triggered by PTSD symptoms (e.g., by increased irritability). Nevertheless, their interrelation suggests that stress resonance may be malleable—similar to PTSD symptoms, which can be alleviated through treatment.

It is important to note that the associations between higher HR and HRV stress resonance and PTSD symptoms were not confirmed in sensitivity analyses, which excluded seven participants who screened positive in a drug test. While initial analysis showed significant associations at an uncorrected threshold, these relationships did not hold after adjusting for multiple comparisons. Since the findings consistently pointed in the same direction, it is likely that the reduced sample size limited statistical power, explaining the lack of significant associations in sensitivity analyses.

We did not find higher cortisol stress resonance to relate to either subjective and autonomic stress resonance, or PTSD symptoms. We explain the lack of association between different stress markers mainly by the multifaceted and complex structure of the stress system [64]. Whereas subjective stress and ANS allow for quick responses within milliseconds (the ANS through synaptic transmissions by SNS and PNS), HPA axis activation occurs in a delayed manner [65]. Stress research therefore regularly encounters this lack of covariance between different dimensions of the stress system [66–68]. Regarding the correlation of cortisol stress resonance with PTSD symptoms, our understanding in earlier studies had been that, compared to the ANS, the delayed cortisol response may be more prone to capture stress resonance and its correlates [16]. Recent studies have shown the opposite, however, suggesting that the prompt reactivity of the ANS may facilitate capturing resonance [15].

Although it has previously been theorized that heightened stress resonance and associated physiological over-activation may lead to detrimental health effects in the long-term [3], empirical evidence for such a link is still sparse. Consistent with this theory, higher levels of HRV resonance during a conflict discussion were shown to predict higher levels of inflammation and greater negative affect reactivity in couples [7]. Our finding of stress resonance being linked to PTSD symptom severity further aligns with this theory, as does the solid body of literature relating personal distress to negative health outcomes [54, 69–72]. However, these findings do not necessarily suggest that a complete lack of stress resonance is desirable. Potential adaptive functions of stress resonance are the mobilization of energy to protect oneself from the stressor at hand, to help those in distress, and to allow for a deeper understanding of others’ experiences [3]. Future studies may wish to consider non-linear relationships between stress resonance and adaptivity.

Our findings have clinical implications, offering the possibility that a training aimed at reducing stress resonance may lead to reductions in PTSD symptom severity in individuals with a history of trauma exposure. By fostering positive affect and strengthening the activation of brain networks implicated in affiliation and reward [73, 74], particularly the training of compassion (defined as the feeling of concern for the suffering of others associated with the motivation to help) may be a resource against the excessive sharing of others’ negative states. Indeed, mindfulness- and compassion-based trainings have recently been shown effective in promoting well-being, trauma recovery and coping with post-migration living difficulties among refugees [75]. These interventions typically target maladaptive self-evaluative emotional responses to trauma and stress by fostering self-compassion, self-acceptance, and nonjudgmental awareness [76–80], and by increasing emotional flexibility [81]—all of which may be beneficial when coping with the suffering of others. Moreover, it seems plausible that learning how to control one’s empathic tendencies also involves the promotion of self-regulation skills, which may enable better coping with first-hand stressors in daily life [82, 83].

Limitations of our study constitute the relatively small sample size, the restricted age range of participants to early and middle adulthood, and the disproportionate low number of female participants, limiting generalizability of our findings. Further, we cannot draw causal conclusions on whether heightened stress resonance preceded or followed the development of PTSD symptoms due to the cross-sectional design of our study. Moreover, we only tested participants’ resonance in opposite-sex dyads and solely in the negative context of stress. Testing whether subjective and physiological resonance is altered also in same-sex dyads and in positive settings, for example when sharing joy in social interactions, may be an important endeavor for future investigations. In addition, we did not assess the year in which our participants were exposed to trauma or the year they arrived in Germany. However, the time elapsed since trauma and the duration spent in the host country may have influenced the association between stress resonance and PTSD symptoms. Finally, our measure of stress resonance bears certain limitations. First, it does not distinguish between observers reacting more and those reacting less strongly than the target, as is captured in the measure of vicarious stress. Second, the timing of the stress peak is not necessarily identical within a dyad, making it impossible to draw conclusions about the attunement of a dyad over time.

In sum, we found that higher subjective and autonomic stress resonance were related to PTSD symptom severity in a sample of individuals from Arabic-speaking countries with varying levels of war-related trauma exposure. This means that the tendency to empathically share the emotional and physiological states of others may be either a precursor, an accompanying factor, or a consequence of the emergence of PTSD. To advance intervention science dedicated to improving refugees’ and migrants’ mental health, efforts should be made to test whether individuals with a history of war-related trauma would benefit from programs aimed at reducing the extent to which they share others’ negative experiences.

## Supplementary information

Supplemental Material

## Data Availability

The data and code used for the current study are available upon request from the authors.

## Footnotes

1 Of note, the two groups were chosen given an initial plan to test whether individuals with war-related trauma (refugees) would differ in empathy from individuals without war-related trauma with a similar cultural background (migrants). Group allocation was based on an initial telephone screening of all participants. However, analyses of a more detailed trauma questionnaire—the Harvard Trauma Questionnaire [26]—revealed that also migrants were exposed to considerable war-related trauma (for discussion, see [27]).
