# Supplementary material for: Adding to the burden: the tendency to resonate with others’ stress is linked to higher PTSD symptom severity in individuals with war-related trauma
Source: Transl Psychiatry. 2025 Aug 30;15:331. doi: 10.1038/s41398-025-03548-4 (PMC12398586; doi:10.1038/s41398-025-03548-4)
Supplement: Supplementary file 1 — Supplemental Material [file 41398_2025_3548_MOESM1_ESM.docx]

**Supplement**

**Table S1. Country of origin of refugees and migrants**

| Country | Refugees (*N* = 35) | Migrants (*N* = 32) |
| --- | --- | --- |
| Syria | 31 | 23 |
| Tunisia | 2 | 0 |
| Yemen | 1 | 0 |
| Lebanon | 1 | 1 |
| Egypt | 0 | 3 |
| Saudi Arabia | 0 | 2 |
| Jordan | 0 | 1 |
| Morocco | 0 | 1 |
| No information | 0 | 1 |

**Table S2. Comparison of vicarious stress markers between smokers and non-smokers of the observer group**

|  | Non-smokers  *N* = 37 | Smokers  *N* = 30 |  |
| --- | --- | --- | --- |
|  | *M* (*SD*) | *M* (*SD*) | *t, p* |
| **Vicarious stress** |  |  |  |
| Subjective stress | 1.46 (1.56) | 1.8 (1.63) | *t* = -0.87, *p* = 0.386 |
| Cortisol ^a^ | 0.04 (0.19) | 0.10 (0.29) | *t* = -1.04, *p* = 0.300 |
| HR ^a^ | -0.00 (0.01) | 0.01 (0.02) | *t* = -1.78, *p* = 0.081 |
| HRV ^a^ | 0.02 (0.15) | 0.01 (0.15) | *t* = 0.14, *p* = 0.887 |

*Note*. HR = heart rate; HRV = heart rate variability.

^a^ Analyses based on log-transformed and winsorized data.

* *p* < .05

**Table S3. Comparison of physiological baseline stress markers between groups**

|  | Refugees  *N* = 35 | Migrants  *N* = 32 | Targets  *N* = 66 |  |
| --- | --- | --- | --- | --- |
|  | *M* (*SD*) | *M* (*SD*) | *M* (*SD*) | *F* (*df*), *p* |
| Subjective stress | 2.11 (1.41) | 1.5 (0.76) | 1.68 (0.84) | *F*(2,130) = 3.43, *p* = 0.036* |
| Cortisol ^a^ | 0.48 (0.21) | 0.43 (0.22) | 0.49 (0.22) | *F*(2,130) = 0.82, *p* = 0.443 |
| HR ^a^ | 1.90 (0.06) | 1.90 (0.06) | 1.90 (0.05) | *F*(2,125) = 0.04, *p* = 0.965 |
| HRV ^a^ | 1.48 (0.29) | 1.57 (0.16) | 1.58 (0.23) | *F*(2,125) = 1.99, *p* = 0.141 |

*Note*. HR = heart rate; HRV = heart rate variability.

^a^ Analyses based on log-transformed and winsorized data.

* *p* < .05

**Table S4. Logistic regression analyses predicting PTSD symptoms: Models including vicarious stress**

|  | *B (SE)* | | *p* | | *OR (95% CI)* | |  | |  | |  |
| --- | --- | --- | --- | --- | --- | --- | --- | --- | --- | --- | --- |
| **Base model (*N* = 67)** | | | | | | | | | | | |
| Intercept | -0.65 (0.32) | | .042 | |  | |  | |  | |  |
| Trauma | 0.81 (0.31) | | .009* | | 2.25 (1.26, 4.34) | |  | |  | |  |
| Sex | 3.03 (0.99) | | .002* | | 20.61 (3.58, 193.02) | |  | |  | |  |
| Age | 0.62 (0.32) | | .054 | | 1.86 (1.02, 3.66) | |  | |  | |  |
| *R*^2^ (Nagelkerke) = 0.336. Model *X*^2^(3) = 19.44, p < .001* | | | | | | | | |  | |  |
| **Models including vicarious stress (*N* = 67)** | | | | | | | | |  | |  |
|  | **Subjective stress** | | | | | | **Cortisol** | | | | |
|  | *B (SE)* | | *p* | | *OR (95% CI)* | | *B (SE)* | | *p* | | *OR (95% CI)* |
| Intercept | -.72 (.35) | | .041 | |  | | -.75 (.35) | | .033 | |  |
| Trauma | .87 (.33) | | .008* | | 2.39 (1.30, 4.83) | | .94 (.34) | | .005* | | 2.55 (1.37, 5.22) |
| Sex | 3.37 (1.05) | | .001* | | 28.99 (4.46, 301.46) | | 3.36 (1.06) | | .002* | | 28.83 (4.53, 320.22) |
| Age | 0.74 (0.35) | | .037 | | 2.09 (1.08, 4.40) | | 0.60 (0.34) | | .077 | | 1.82 (0.97, 3.71) |
| Vicarious stress | 0.84 (0.34) | | .014 | | 2.32 (1.23, 4.82) | | .64 (.31) | | .038 | | 1.89 (1.07, 3.66) |
| Trauma*Vicarious stress | 0.01 (0.35) | | .983 | | 1.01 (0.52, 2.15) | | -.05 (.35) | | .879 | | 0.95 (0.47, 1.99) |
| Model fit | *R*^2^ (Nagelkerke) = 0.436. Model *X*^2^(5) = 26.45, *p* < .001* | | | | | | *R*^2^ (Nagelkerke) = 0.408. Model *X*^2^(5) = 24.41, *p* < .001* | | | | |
| Delta model fit | *X*^2^(2) = 7.01, *p* = .030 | | | | | | *X*^2^(2) = 4.97, *p* = .083 | | | | |
| **Base model (subsample with complete autonomic data, *N* = 64)** | | | | | | | | | | | |
|  | *B (SE)* | *p* | | *OR (95% CI)* | |  | |  | |  | |
| Intercept | -.59 (.32) | .067 | |  | |  | |  | |  | |
| Trauma | .77 (.31) | .013 | | 2.16 (1.21, 4.14) | |  | |  | |  | |
| Sex | 2.81 (1.00) | .005* | | 16.69 (2.82, 158.96) | |  | |  | |  | |
| Age | 0.59 (0.32) | .062 | | 1.81 (1.00, 3.53) | |  | |  | |  | |
| *R*^2^ (Nagelkerke) = 0.308. Model *X*^2^(3) = 16.80, p < .001* | | | | | | | | | | | |
| **Models including vicarious stress (*N* = 64)** | | | | | | | | | | | |
|  | **HR** | | | | | **HRV** | | | | | |
|  | *B (SE)* | *p* | | *OR (95% CI)* | | *B (SE)* | | *p* | | *OR (95% CI)* | |
| Intercept | -.64 (.33) | .050 | |  | | -.60 (.32) | | .063 | |  | |
| Trauma | .67 (.32) | .038 | | 1.96 (1.06, 3.85) | | .74 (.32) | | .019 | | 2.11 (0.28, 1.01) | |
| Sex | 3.00 (1.12) | .008* | | 20.11 (2.74, 251.95) | | 2.86 (1.06) | | .007* | | 17.45 (2.69, 11.46) | |
| Age | 0.67 (0.33) | .045 | | 1.95 (1.05, 3.94) | | 0.63 (0.33) | | .058 | | 1.88 (1.01, 3.80) | |
| Vicarious stress | 0.33 (0.32) | .291 | | 1.39 (0.76, 2.67) | | 0.12 (0.30) | | .697 | | 1.12 (0.62, 2.06) | |
| Trauma*Vicarious stress | 0.28 (0.32) | .392 | | 1.32 (0.79, 2.59) | | 0.10 (0.40) | | .806 | | 1.10 (0.51, 2.49) | |
| Model fit | *R*^2^ (Nagelkerke) = 0.336. Model *X*^2^(5) = 18.57, *p* = .002* | | | | | *R*^2^ (Nagelkerke) = 0.311. Model *X*^2^(5) = 17.00, *p* = .004* | | | | | |
| Delta model fit | *X*^2^(2) = 1.77, *p* = .413 | | | | | *X*^2^(2) = 0.20, *p* = .906 | | | | | |

*Note*. HR = heart rate; HRV = heart-rate variability (indexed via RMSSD)

* *p* < .0125

**Table S5. Logistic regression analyses predicting PTSD symptoms (participants with positive drug screen excluded)**

|  | *B (SE)* | | *p* | | *OR (95% CI)* | |  | |  | |  |
| --- | --- | --- | --- | --- | --- | --- | --- | --- | --- | --- | --- |
| **Base model (*N* = 60)** | | | | | | | | | | | |
| Intercept | -0.68 (0.35) | | .054 | |  | |  | |  | |  |
| Trauma | 0.79 (0.34) | | .019 | | 2.21 (1.17, 4.51) | |  | |  | |  |
| Sex | 3.09 (1.02) | | .003* | | 21.98 (3.60, 219.31) | |  | |  | |  |
| Age | 0.69 (0.36) | | .055 | | 2.00 (1.02, 4.28) | |  | |  | |  |
| *R*^2^ (Nagelkerke) = 0.362. Model *X*^2^(3) = 18.95, p < .001 | | | | | | | | |  | |  |
| **Models including stress resonance (*N* = 60)** | | | | | | | | |  | |  |
|  | **Subjective stress** | | | | | | **Cortisol** | | | | |
|  | *B (SE)* | | *p* | | *OR (95% CI)* | | *B (SE)* | | *p* | | *OR (95% CI)* |
| Intercept | -0.66 (0.39) | | .095 | |  | | -0.68 (0.35) | | .055 | |  |
| Trauma | 0.71 (0.37) | | .059 | | 2.03 (1.00, 4.48) | | 0.84 (0.35) | | .017 | | 2.31 (1.20, 4.89) |
| Sex | 2.71 (1.14) | | .017 | | 15.07 (2.00, 203.81) | | 3.03 (1.02) | | .003* | | 20.65 (3.83, 206.06) |
| Age | 0.88 (0.41) | | .033 | | 2.42 (1.14, 5.94) | | 0.59 (0.37) | | .109 | | 1.81 (0.90, 3.93) |
| Stress resonance | 1.00 (0.40) | | .012* | | 2.72 (1.32, 6.42) | | 0.14 (0.33) | | .680 | | 1.14 (0.60, 2.23) |
| Trauma*Stress resonance | 0.42 (0.42) | | .318 | | 1.52 (0.68, 3.65) | | 0.27 (0.30) | | .363 | | 1.31 (0.75, 2.56) |
| Model fit | *R*^2^ (Nagelkerke) = 0.503. Model *X*^2^(5) = 28.35, *p* < .001 | | | | | | *R*^2^ (Nagelkerke) = 0.383. Model *X*^2^(5) = 20.29, *p* = .001 | | | | |
| Delta model fit | *X*^2^(2) = 9.41, *p* = .009** | | | | | | *X*^2^(2) = 1.34, *p* = .511 | | | | |
| **Base model based on sub-sample with complete HR/V resonance data (*N* = 56)** | | | | | | | | | | | |
|  | *B (SE)* | *p* | | *OR (95% CI)* | |  | |  | |  | |
| Intercept | -.61 (.35) | .088 | |  | |  | |  | |  | |
| Trauma | .74 (.34) | .029 | | 2.09 (1.11, 4.26) | |  | |  | |  | |
| Sex | 2.69 (1.05) | .010* | | 14.76 (2.26, 154.19) | |  | |  | |  | |
| Age | 0.66 (0.35) | .060 | | 1.93 (1.01, 4.07) | |  | |  | |  | |
| *R*^2^ (Nagelkerke) = 0.318. Model *X*^2^(3) = 15.25, *p* = .002** | | | | | | | | | | | |
| **Models including stress resonance (*N* = 56)** | | | | | | | | | | | |
|  | **HR** | | | | | **HRV** | | | | | |
|  | *B (SE)* | *p* | | *OR (95% CI)* | | *B (SE)* | | *p* | | *OR (95% CI)* | |
| Intercept | -0.67 (0.39) | .088 | |  | | -0.84 (0.42) | | .044 | |  | |
| Trauma | 0.75 (0.38) | .046 | | 2.09 (1.10, 4.30) | | 0.58 (.39) | | .134 | | 1.79 (0.86, 4.04) | |
| Sex | 3.27 (1.21) | .007* | | 22.04 (2.97, 280.32) | | 3.69 (1.35) | | .006* | | 39.88 (3.79, 850.17) | |
| Age | 0.98 (0.42) | .019 | | 2.45 (1.25, 5.50) | | 1.25 (0.49) | | .011* | | 3.48 (1.46, 10.33) | |
| Stress resonance | 0.93 (0.38) | .015 | | 2.53 (1.30, 5.51) | | 1.11 (0.47) | | .018 | | 3.03 (1.33, 8.59) | |
| Trauma*Stress resonance | 0.40 (0.34) | .238 | | 1.66 (0.89, 3.45) | | 0.37 (0.38) | | .340 | | 1.44 (0.67, 3.13) | |
| Model fit | *R*^2^ (Nagelkerke) = 0.456. Model *X*^2^(5) = 23.38, *p* < .001* | | | | | *R*^2^ (Nagelkerke) = 0.472. Model *X*^2^(5) = 24.41, *p* < .001* | | | | | |
| Delta model fit | *X*^2^(2) = 8.13, *p* = .017 | | | | | *X*^2^(2) = 9.16, *p* = .010* | | | | | |

*Note*. HR = heart rate; HRV = heart-rate variability (indexed via RMSSD)

* *p* < .0125
